# Supplementary material for: A set of multi-entry identification keys to African frugivorous flies (Diptera, Tephritidae)
Source: Zookeys. 2014 Jul 24;(428):97–108. doi: 10.3897/zookeys.428.7366 (PMC4143993; doi:10.3897/zookeys.428.7366)
Supplement: Supplementary material 4 — Key to Capparimyia [file zookeys-428-097-s004.zip › SF4_ZooKeys_key to Capparimyia/lucid_player/help/help/what_does_the_lucid3_builder_do_.htm]

About the Lucid3 Builder


# About the Lucid3 Builder

Lucid3 keys are created in the Lucid3 Builder. Using the Builder, an
expert in a group of entities (plants, animals, diseases, minerals, archaeological
artefacts etc) creates interactive, random-access keys that can be deployed
over the World Wide Web or CD.

The basic elements of a Lucid3 key are -

- a list of entities
- a list of features and states that may be used
  to describe those entities
- a matrix of score data for the features associated
  with each of the entities
- various attachments (images, web pages etc) for
  the entities and features, to provide extra information to users.

The Lucid3 Builder provides all the tools necessary to create the entity
and feature lists, encode the score data, and attach information files
to items.

For more information on the Lucid3 Builder, visit the Lucidcentral website
at www.lucidcentral.org.
